# The antitumour effects of eudesmin on lung cancer by inducing apoptosis via mitochondria-mediated pathway in the tumour cells

**DOI:** 10.1080/13880209.2017.1401647

**Published:** 2017-11-24

**Authors:** Li-Li Jiang, Bai-Rong Sun, Chao Zheng, Gui-Lun Yang

**Affiliations:** a Department of Acupuncture and Moxibustion, Linyi People's Hospital, Linyi, PR China;; b Department of Traditional Chinese Medicine, Linyi People's Hospital, Linyi, PR China;; c Department of Orthopedic, Linyi People's Hospital, Linyi, PR China;; d Department of Medical Imaging, Linyi People's Hospital, Linyi, PR China

**Keywords:** A549 cells, Akt/JNK signalling pathway, molecular mechanisms

## Abstract

**Context:** Limonoids possess broad range of biological activities, including antitumour, antimicrobial and antioxidant activities, etc. Eudesmin (EDN) is a type of limonoid which also possesses various activities. However, there is no report on the antitumour lung cancer (LC) activities of this compound.

**Objective:** The present study investigates the antitumour effects of EDN and its potential molecular mechanisms.

**Materials and methods:** The *in vitro* antitumour effects of EDN on LC A549 cells were evaluated by using MTT assay. The *in vivo* antitumour effects were investigated on a xenograft athymic nude mouse model. The mice were administered orally with EDN (10, 20 and 40 mg/kg) once daily for 28 days. Effects of EDN on apoptosis-related or signalling proteins (Bcl-2, Bax, caspase-3, caspase-9, P53, Akt and JNK) were assayed by western blot analysis.

**Results:** EDN showed significant inhibitory effects on the growth of LC A549 cells *in vitro* with the half maximal inhibitory concentration (IC_50_) of 18.3 μM. By treating with EDN (10, 20 and 40 μM), expression of caspase-3, caspase-9, Bax, P53 and phosphorylated JNK in A549 cells were significantly upregulated, whereas expression of Bcl-2 and Akt phosphorylation were significantly down-regulated. Interestingly, EDN-induced apoptosis could be attenuated by JNK inhibitor. In addition, *in vivo* experiments also indicated EDN (10, 20 and 40 mg/kg) had significant antitumour effects (*p* < 0.01) on nude mice.

**Conclusions*:***Overall, the results indicated that EDN possesses significant antitumour effects on LC and the possible mechanism might be related to induction of mitochondria-mediated apoptosis.

## Introduction

Worldwide, lung cancer (LC) is one of the most commonly occurring cancers in humans, there are more than one million deaths every year (Zajdel et al. 2015). Non-small cell lung cancer (NSCLC) is the most common type of LC which accounts for about 75–85% (Zhu et al. 2015). Depends on the histological type and the stage of NSCLC, the treatment for this cancer often employs combined methods, including radiotherapy, chemotherapy and surgery (Tezuka et al. 2012; Harada et al. 2013). The current standard first-line treatment for advanced NSCLC patients is platinum-based chemotherapy, which is not considered satisfactory to patients with the median survival time of 8–10 months (Motohashi et al. 2011). Therefore, exploring new drugs to improve the efficiency of NSCLC treatment and reducing the toxic side effects have been urgent problems.

Natural products have drawn increasing attention because many important cancer preventive and therapeutic agents have been developed from them (Wang et al. 2014). Eudesmin (EDN) is a lignan which has been isolated from different plant families, including Apiaceae, Rutaceae, Ochnaceae and Magnoliaceae (Raimundo et al. 2009). Various investigations have shown EDN possesses broad range of biological activities, such as cytotoxic, antibacterial, antifungal, inhibitory effects on tumour necrosis factor-α production, anticonvulsant, sedative and vascular relaxation effects (Raimundo et al. 2009; Liu et al. 2015). However, to the best of our knowledge, there is no report on the antitumour activities of this compound on LC.

In the present study, the antitumour effect of EDN was investigated in human lung carcinoma A549 cells *in vitro* and tumour cell transplanted mice *in vivo*. Furthermore, apoptosis related proteins including P53, Bcl-2, Bax, caspase-9, caspase-3, Akt and JNK were evaluated to explore the mechanism of the antitumour effects.

## Materials and methods

### Chemicals and reagents

Eudesmin (purity >98%) was obtained from Chengdu Herbperity Co., Ltd (Chengdu, China). 1-(4,5-Dimethylthiazol-2-yl)-3,5-diphenyl-formazan (MTT) was purchased from Sigma-Aldrich (St. Louis, MO). The primary monoclonal antibodies of P53, Bcl-2, Bax, caspase-9, caspase-3, Akt, JNK and horseradish peroxidase-conjugated secondary antibodies were obtained from Beyotime Biotechnology (Shanghai, China). RPMI1640 culture medium and foetal bovine serum (FBS) were purchased from American Hyclone Company (Logan, UT). All other chemicals and reagents used in this study were of analytical grade.

### Cell culture

Human lung carcinoma A549 cells were obtained from the Institute of Biochemistry and Cell Biology, Chinese Academy of Sciences (Shanghai, China). They were maintained in RPMI-1640 culture medium supplemented with 10% FBS, 100 U/mL penicillin, and 100 μg/mL streptomycin, and incubated at 37 °C in 5% CO_2_/95% air.

### The effect of EDN on cell proliferation

The effect of EDN on A549 cell proliferation was assayed by using MTT assay according to a previous study (Lin et al. 2016). Briefly, A549 cells were seeded into 96-well plate at the concentration of 1 × 10^4^ cells/mL and incubated overnight. Then, the cells were incubated with different concentrations of PSDP (2.5, 5, 10, 20, 40 and 80 μM) for 24 h. MTT (5 mg/mL, 20 μL) was added to each well and cultured for 4 h. After MTT solution was removed, DMSO (150 μL) was added and oscillated for 15 min. The absorbance was measured at 570 nm using a microplate reader (Bio-Rad, Hercules, CA). The inhibition of cell proliferation was calculated by the following formula:

$$\text{Inhibition }\left(\%\right)=\left(A_{0}-\;A_{\text{t}}\right)/A_{0}\times \text{ 1}00$$

where *A*_0_ is the absorbance of the control group and *A*_t_ is the absorbance of EDN treated group.

### Animals

Male athymic nude mice (nu/nu) aging 4–6 weeks (18 ± 1 g) were purchased from SLAC Laboratory Animal Co., Ltd. (Shanghai, China). All mice were housed in a temperature of 21 ± 2 °C, relative humidity (65 ± 10%) and a 12 h light/dark cycle environment, and fed with food and water *ad libitum*. The mice were acclimatized for one week before experiment. Animal experiment was carried out in accordance with Canadian Council on Animal Care (CCAC) guidelines and the experiments were approved by the Animal Ethics Committee of Linyi People's Hospital (Linyi, China).

### *In vivo* antitumour efficacy study

Subcutaneous tumour model was established in athymic nude mice by inoculation of A549 cells (1 × 10^6^/0.2 mL) into the dorsal flank of the mouse (Razi et al. 2015). When tumours grew to approximately 4–5 mm in diameter, animals were randomly divided into four groups (*n* = 10): the control group (distilled water), different EDN groups (10, 20 and 40 mg/kg). The mice were then administered orally once daily for 28 days, and tumour volumes were measured by digital calliper during this period. Tumour volume was calculated by a standard formula: (width^2^×length)/2. After 24 h of the last administration, the mice were sacrificed, and the tumours weights and body weights were measured.

### Western blot analysis

Western blot analysis was performed as previously reported (Katanasaka et al. 2014). The proteins of the cells or tumour tissues were extracted. An equal amount of proteins (50 μg) in each sample was separated by 10% sodium dodecyl sulphate-polyacrylamide gel electrophoresis (SDS-PAGE). After electrophoresis, the proteins were transferred to a polyvinylidene difluoride (PVDF) membrane and blocked with 5% skim-milk in TBST (20 mM Tris–HCl, 0.9% NaCl and 0.1% Tween-20). The membrane was incubated overnight at 4 °C with primary antibodies, and then with horseradish peroxidase-conjugated secondary antibodies for 1 h at room temperature. Protein bands were visualized by using chemiluminescence reagents.

### Statistical analysis

All data were expressed as means ± standard deviation (SD). Statistical analysis was performed by Student’s *t*-test using SPSS software version 17.0 (SPSS Inc., Chicago, IL). Differences of groups were considered statistically significant when *p* < 0.05.

## Results

### *In vitro* antitumour activity of EDN

**Figure 1.**
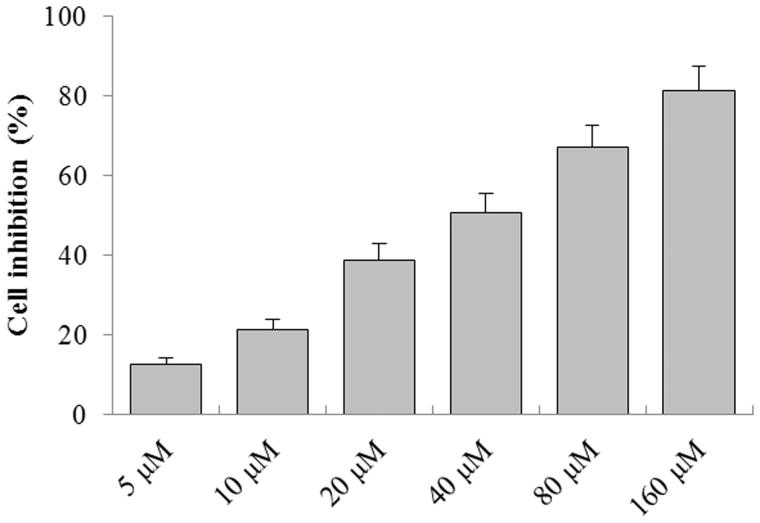
The antitumour effect of EDN on A549 cells.

As shown in Figure 1, the *in vitro* antitumour activity of EDN was assayed by MTT in a concentration range from 2.5 to 80 μM. As a result, EDN showed significant inhibitory effect on the growth of A549 cells with the increase of concentration. The half maximal inhibitory concentration (IC_50_) of EDN on A549 cell growth was 18.3 μM. Thus, the concentrations of 10, 20 and 40 μM were selected for further experiments.

### Effects of EDN on the expression of P53, Bcl-2, Bax,caspase-9 and caspase-3 in A549 cells

**Figure 2.**
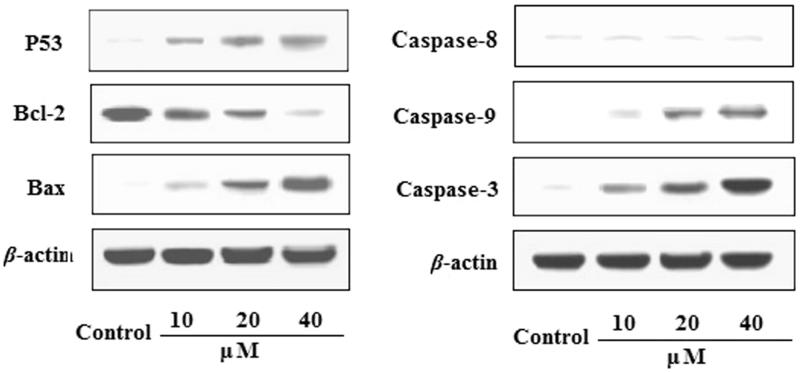
Effects of EDN on the expression of P53, Bcl-2, Bax, caspase-9 and caspase-3 in A549 cells.

The effects of EDN on the expression of P53, Bcl-2, Bax, caspase-9, caspase-8 and caspase-3 proteins in A549 cells are shown in Figure 2. The results indicated that the expression of Bax protein was significantly upregulated, while the expression of Bcl-2 protein was significantly downregulated by treating with EDN (10, 20 and 40 μM) in concentration-dependent manners. The expression of caspase-3 and caspase-9 proteins in EDN-treated group was significantly upregulated compared with control group. In addition, EDN also significantly upregulated the expression of P53 protein with a concentration-dependent manner. The results indicated that EDN can induce apoptosis in lung carcinoma A549 cells, and the mechanisms may be involved in mitochondria-mediated apoptosis pathway. Furthermore, EDN showed no significant effects on the expression of caspase-8 compared with the control cells, indicating that EDN induced apoptosis was not via death-receptor-mediated pathway.

### Effects of EDN on the expression of Akt and JNK in A549 cells

**Figure 3.**
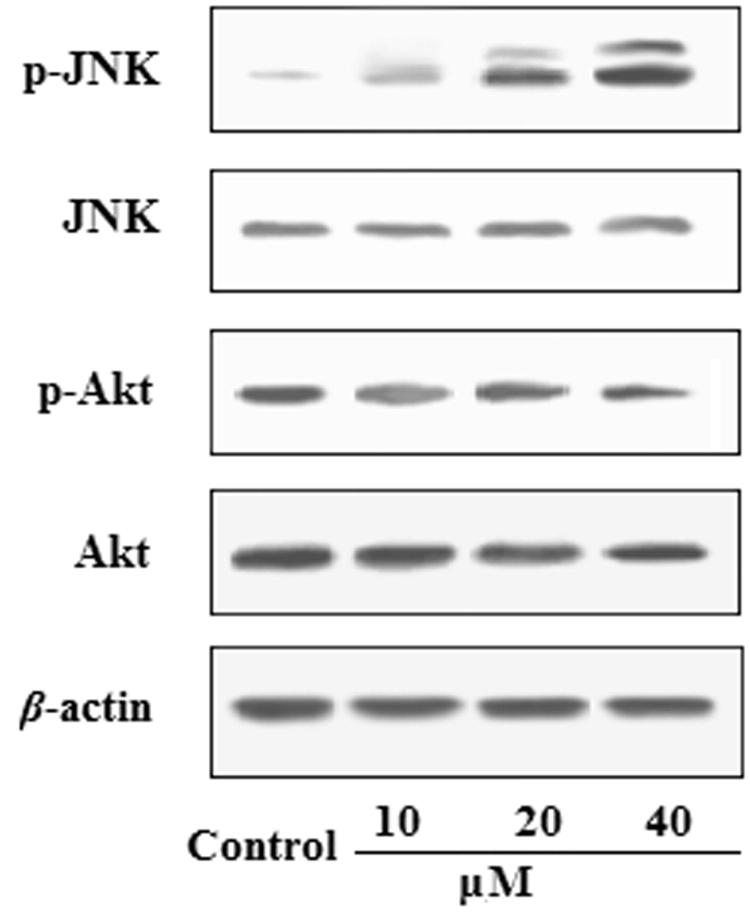
Effects of EDN on the expression of Akt and JNK in A549 cells.

To investigate whether the pro-apoptosis effects of EDN through the cell JNK/Akt signal pathway, the expression of Akt and JNK proteins in A549 cells was evaluated. As shown in Figure 3, JNK phosphorylation levels significantly increased, whereas Akt phosphorylation levels were decreased after treatment with EDN at the concentrations of 10, 20 and 40 μM in A549 cells.

### Inactivation of JNK reduced EDN-induced apoptosis in A549 cells

**Figure 4.**
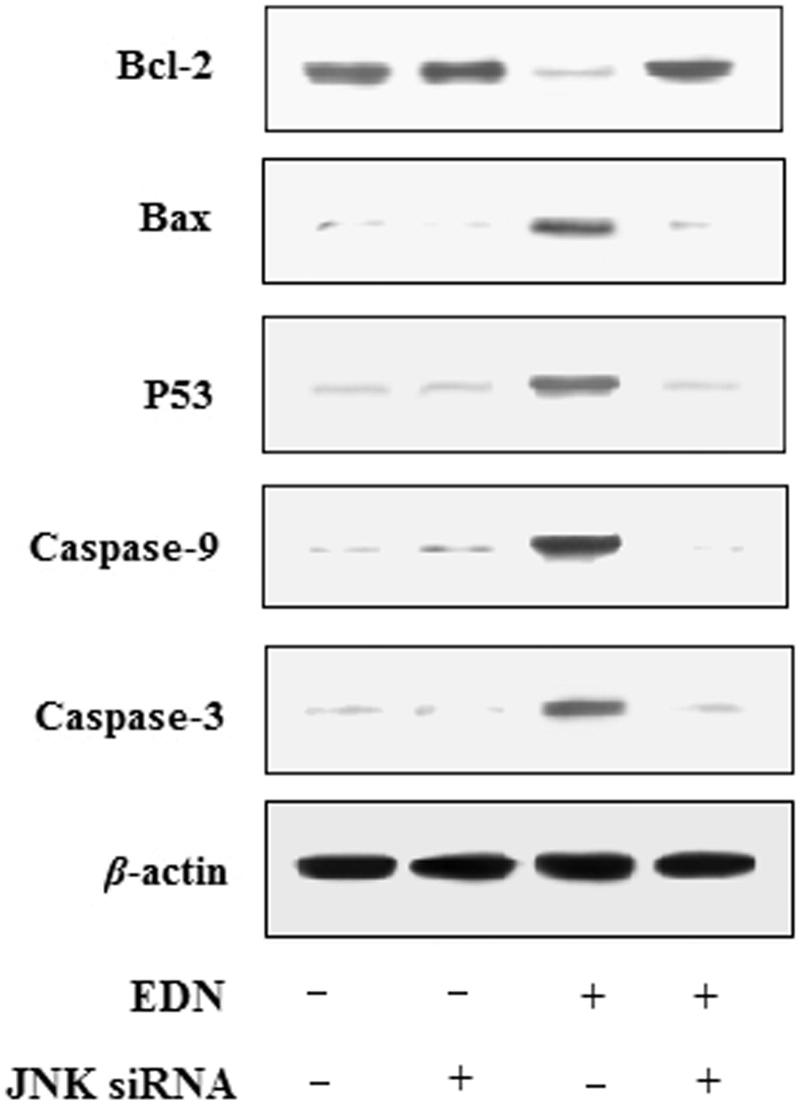
Effects of JNK siRNA on expression of apoptosis-related proteins in A549 cells.

To further determine whether regulation of JNK was required in EDN-induced apoptosis, the A549 cells were treated with 40 μM EDN in the presence or absence of the JNK siRNA. The results are shown in Figure 4, the expression of caspase-3, caspase-9, P53 and Bax was reduced, while the expression of Bcl-2 was increased in JNK siRNA-transfected A549 cells. Therefore, the results showed that the activation of JNK played an vital role in EDN-induced apoptosis.

### *In vivo* antitumour activity of EDN

**Figure 5.**
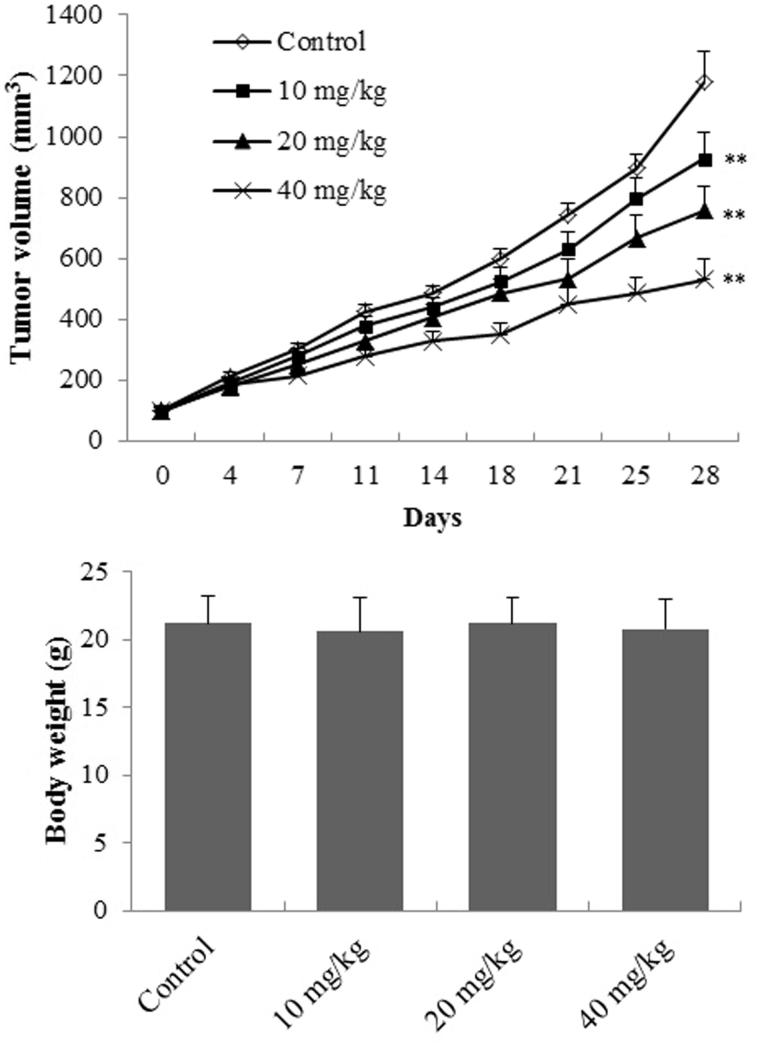
The effect of EDN on tumour weight and body weight of mice. **p < 0.01, compared with control group.

The effect of EDN on tumour weight and the body weight in A549 cells transplanted mice is shown in Figure 5. The tumour volume of EDN treated mice was significantly decreased (*p* < 0.01) at the doses of 10, 20 and 40 mg/mL compared with the mice in control group. However, EDN showed no significant effect on the body weight of mice (*p* > 0.05) after oral administration for 28 days compared with the control mice, indicating that EDN had no obvious toxicity.

### Effects of EDN on the expression of P53, Bcl-2, Bax,caspase-9, caspase-3 and JNK in tumour tissues

**Figure 6.**
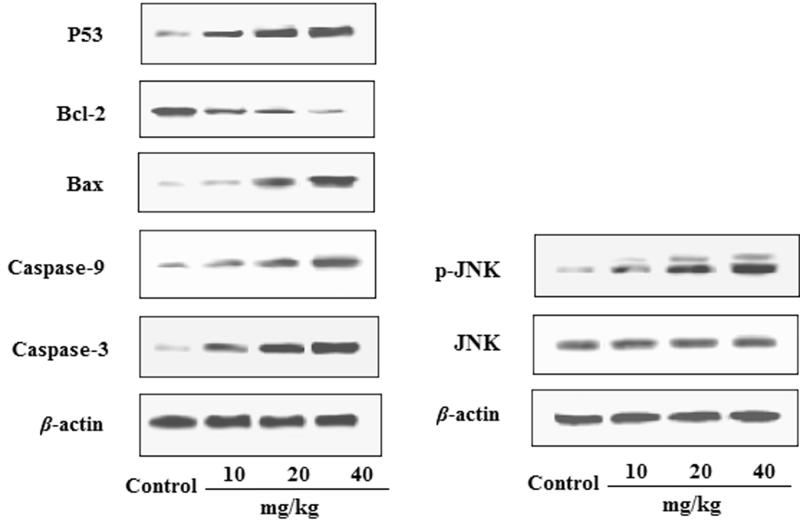
Effects of EDN on the expression of P53, Bcl-2, Bax, caspase-9, caspase-3 and JNK in A549 cells.

The effects of EDN on the expression of P53, Bcl-2, Bax, caspase-9, caspase-3 and JNK in tumour tissues are shown in Figure 6. The results indicated that by treating with EDN, the expression of Bax, caspase-3, caspase-9 and P53 proteins was significantly up-regulated, whereas the expression of Bcl-2 protein was significantly down-regulated. In addition, the expression of JNK phosphorylation was also significantly upregulated by treating EDN with a dose-dependent manner. These findings confirmed the antitumour activity of EDN *in vivo*, and also confirmed that the EDN-induced apoptosis is associated with the mitochondria-mediated apoptosis pathway.

## Discussion

Lung cancer is an intractable disease and still remains a leading cause of cancer death in the world. However, current antitumour agents often have serious side effects (Park et al. 2011). Thus, it is urgent to search for agents with higher bioactivity and lower toxicity for treating LC. Lignans is a group of diphenolic compounds where the C_6_–C_3_ carbons are bound by the C_8_ central carbon. They are widely distributed in more than 70 families of vascular plants and have been isolated from different parts of plants, such as roots, stems, rhizomes, leaves, seeds and fruits, etc. (Jiang et al. 2015). Although lignans were neither non-nutrient nor non-caloric, they have attracted increasing attention because of their various biological effects, including antitumour, antiviral and antioxidant activities (Chun et al. 2014). In this study, EDN was proved to possess significant antitumour effects on LC A549 cells both *in vitro* and *in vivo*, demonstrating that it had the potential to develop into antitumour drugs for LC in the future.

Apoptosis, known as programed cell death, has been recognized as an ideal method of cancer therapy, and is controlled by genes and a series of enzymes (Tai et al. 2016). The expression of Bcl-2 family proteins plays an important role in the process of apoptosis, which involved in mitochondrial pathway. A high ratio of the Bcl-2 family proteins (Bax/Bcl-2) contributed to the apoptosis-promotion (Chen et al. 2016). Caspases are the key proteins that modulate the apoptotic response. The activation of caspase-3 is one of the most common involvements in process of the apoptosis in various cell types, which can be commonly activated by caspase-9 (Li et al. 2013). Furthermore, expression of P53 protein also plays pivotal roles in the initiation and execution of apoptosis (Ding et al. 2012; Peng et al. 2017). In the present study, the expression of caspase-3, caspase-9, Bax and P53 proteins were significantly upregulated, whereas the expression of Bcl-2 protein was significantly downregulated by treating with EDN. The results indicated that the antitumour effect of EDN was closely related to induction of apoptosis in A549 cells.

It is reported that the MAPK pathway plays important roles in cell proliferation, apoptosis and differentiation, and can be activated by several stimuli (Gao et al. 2011). JNK is one of the major subfamilies of MAPK pathway which can activate Bax, cytochrome c and the onset of mitochondrial apoptosis (Yu et al. 2017). Akt is a serine–threonine kinase which is critical for regulating cell survival and blocking apoptosis, and acts as a hub in regulating multi-pathways (Gao et al. 2011). In addition, Akt also can repress JNK pathways to inhibit apoptosis (Zhang et al. 2016). In this study, JNK phosphorylation levels significantly increased, whereas Akt phosphorylation levels were decreased after treatment with EDN. To further explore the role of JNK in mitochondria-mediated EDN-induced apoptosis, a JNK inhibitor was used. The results indicated that EDN-induced apoptosis was significantly attenuated by the JNK inhibitor. Thus, the results indicated that the apoptosis induced by EDN was via the Akt/JNK signalling pathway.

## Conclusions

In conclusion, the present study proved that EDN possessed marked antitumour effects on LC A549 cells both *in vitro* and *in vivo*. The mechanisms of the antitumour effects might be closely related to the induced apoptosis via mitochondria-mediated pathway in the tumour cells. EDN has potentials to be developed into antitumour drugs for the treatment of LC.
